# A Sting Operation: Risk Assessment and Venom Expenditure by Arizona Bark Scorpions (*Centruroides sculpturatus*) in a Defensive Context

**DOI:** 10.3390/toxins17040198

**Published:** 2025-04-13

**Authors:** Lindsay A. Marston, Gerad A. Fox, Kim Y. Hung, Shannon J. Delo, William K. Hayes

**Affiliations:** 1 Department of Earth and Biological Sciences, School of Medicine, Loma Linda University, Loma Linda, CA 92354, USA; lindsmarston@gmail.com (L.A.M.); gfox411@gmail.com (G.A.F.); marcysj@masters.edu (S.J.D.); 2 Coachella Valley Mosquito and Vector Control District, 43-420 Trader Pl, Indio, CA 92201, USA; khung@cvmosquito.org

**Keywords:** defensive behavior, antipredator behavior, predator avoidance behavior, threat avoidance, venom metering, invasive species, predator simulation, sexual dimorphism, wet stings

## Abstract

Scorpion antipredator behavior incorporates risk assessment that informs decision-making and venom usage. We quantified antipredator behaviors of the clinically significant Arizona bark scorpion (*Centruroides sculpturatus*) in their natural environment using exposure to two stimuli: a freshly thawed laboratory mouse (*Mus musculus*) and a membrane-covered glass beaker. We videotaped and compared envenomation behaviors between sexes (females, gravid females, and males), across sizes, and between animal orientations (on vertical or horizontal substrates). Results failed to show consistent support for any of our four hypotheses. Females (especially gravid females) were no more likely than males to exhibit higher levels of stinging and venom expenditure. Scorpions on horizontal surfaces compared to those on vertical surfaces, and larger scorpions compared to smaller ones, were likewise no more likely to exhibit higher levels of responsiveness. Mice were more likely to be stung than the membrane-covered beaker, but with fewer and briefer stings, suggesting the scorpions did not attempt to deliver more venom into the mice. Thus, we discerned no clear patterns in risk assessment, stinging, and venom use associated with sex, substrate orientation, body size, or threat stimuli. These findings contrasted with those of several prior laboratory studies. Variation from unaccounted environmental variables may have obfuscated divergent behavioral tactics. Nevertheless, the behaviors we document here provide insights on the range of defensive behaviors exhibited by *C. sculpturatus* under natural environmental conditions, including the frequency of dry stings (11.8%) to the membrane-covered beakers.

## 1. Introduction

Risk assessment and subsequent adjustments of defensive behavior enable animals that possess venom to use it strategically [1,2,3,4,5,6,7,8], as it is a metabolically costly commodity [8,9,10]. Venomous animals often make decisions about deploying their venom and may modulate the quantity or composition of venom expulsed. Some will even target their venom toward a vulnerable region of their prey. The capacity to control or modulate venom quantity comprises venom metering [1,2,3,5] or venom optimization [4,6], and has been documented in a number of animal groups. Most work on venom metering has focused on snakes [1,2,3,5,11]. However, a number of invertebrates also exhibit venom metering. Among arthropods, several spider species [4,7,12], buthid scorpions in general [13,14,15,16,17,18,19], the hadrurid scorpion *Hadrurus arizonensis* [15,20,21], the vaejovid scorpion *Smeringurus mesaensis* [15,17], and the urodacid scorpion *Urodacus yaschenkoi* [22] are capable of behaviorally modulating venom expenditure, at least in response to different levels of threat.

Sexual differences in defensive behavior have been identified in scorpions, often with consideration of sexual dimorphism in overall size or individual body components. In *Centruroides vittatus*, for example, females have more massive mesosomas, shorter and wider metasoma segments, and shorter legs than males [23]. Differences in morphology were associated with sexual differences in defensive behavior, wherein females were more likely to attack with a shorter latency to react, stung more rapidly and more often, and sprinted slower than males when escape was attempted [23,24]. The females also expended more venom (though similar to males if adjusted for body size), and synthesized venom that was less painful to rodent predators [25]. The relationship seen in *C. vittatus* was reversed in the sexually dimorphic Brazilian scorpion *Tityus pusillus*, with females more likely to flee than males [26]. In this species, no difference in defensive stinging with respect to sex was detected at either low or high threat levels [27].

Habitat and refuges therein can influence the defensive behaviors of animals. Some animals tolerate closer encounters before fleeing when the level of risk is reduced, such as when refuge is close [28,29,30]. Among scorpions, the speed with which defensive chelae pinching occurred in *Vaejovis carolinianus* changed with refuge availability and females stung more frequently in the presence of a refuge [31]. *Centruroides vittatus* may climb vegetation primarily for predator avoidance and secondarily for foraging [32], but prefers foraging in vegetation rather than on the ground, with vegetation type varying among scorpions of different size [33]. Escape trajectory options increase on a vertical surface because there is the option to drop [34,35], although this may be a last resort as it increases the chance of injury. Slender branches allow the prey to increase vertical distance from the predator when fleeing toward the canopy, toward the base, or by falling, whereas lateral trajectories utilize the branch itself as a barrier but limit the distance of flight. Vegetation may offer as much refuge (in the form of cracks, bark, and foliage) as horizontal surfaces on the ground (in the form of cracks, burrows, and substrate), but provide one additional escape trajectory, dropping [34], which might influence the likelihood of engaging the predator before fleeing. No research has described any influence that horizontal or vertical substrate orientation may have on scorpion defensive behaviors, although other species have been assessed. When climbing trees, for example, lizards accelerate faster but pause more frequently when escaping on a vertical tree trunk compared to horizontal surfaces [35].

Size-related variation in morphology and defensive behavior has also been demonstrated in scorpions. These animals employ defensive tactics that are most successful with respect to the morphology and effectiveness of their weapons [36,37,38]. Younger, smaller scorpions possess smaller telsons with reduced venom gland capacity, a smaller aculeus (stinger) to penetrate the target, and a shorter metasoma to generate thrust during stinging. Scorpion venom volume, venom flow, and strike efficiency are also dictated by the physics of size [15]. *Tityus pusillus* showed varying defensive behaviors between juveniles and the much larger adults, with juveniles more likely to flee during the restriction of various body parts [26]. Smaller specimens of *H. arizonensis*, in contrast, resorted more quickly to defensive stinging than adults [21]. In a comparative study, expelled venom varied across five scorpion species, with smaller species exhibiting stings of longer duration presumably to facilitate increased venom expulsion [15]. Scorpion size may influence other behaviors, as size is associated with perceived risk in certain microhabitats. Juveniles of *C. vittatus*, for example, avoid cacti that have a large population of adult scorpions [39].

Bark scorpions in the genus *Centruroides* are called such for their use of several microhabitats including trees with loose bark that offer refuge [40,41]. Microhabitats such as trees, rocky outcrops, and walls that offer vertical surfaces and places for refuge could influence the defensive behavior of these largely lithophilic scorpions. Information on how substrate orientation influences *C. sculpturatus* defensive behavior and venom metering may be useful for landowners who regularly encounter this medically relevant species throughout landscaped properties in portions of the Sonoran Desert region within their range, which includes much of Arizona and narrow adjoining areas of California, Nevada, New Mexico, and Utah.

In this study, we conducted two field experiments on bark scorpions of an introduced urban population in southern California to test their abilities to assess risk, alter antipredator behavior, and adjust venom expenditure under unmanipulated environmental conditions. We tested the following hypotheses by presenting threatening targets—either a dead laboratory mouse (*Mus musculus*) or a membrane-covered beaker—to adult *C. sculpturatus* in their habitat and videotaping their responses:

**H1.**  *Females, because of their relatively greater mass and stouter bodies, should rely more on stinging and venom use, and less on flight, than males, as reported for C. vittatus [23,24]. Moreover, gravid females encumbered by developing embryos should rely even more on stinging and venom use than non-gravid females. We predicted that sting use, number of stings before fleeing, duration of first sting, and proportion of wet stings should be in the order of gravid females > non-gravid females > males, whereas latency to sting should be in the reverse order.*

**H2.**  *Scorpions on horizontal substrates rely more on stinging and venom than those on vertical substrates because the latter offer more escape trajectories, reducing the risk of capture [28,29,30,34,35]. We predicted that scorpions on horizontal surfaces would be less inclined to flee, and therefore more likely to issue a sting and deliver more stings with reduced latency, greater duration, and a higher percentage of wet stings.*

**H3.**  *Smaller scorpions have less capacity to deliver stings and venom than adults [15,42], and are therefore more likely to flee than defend themselves by stinging. We predicted a positive relationship between scorpion size and sting use, number of stings, duration of first sting, and proportion of wet stings, as well as a negative correlation between scorpion size and latency to sting.*

**H4.**  *Scorpions exhibit elevated defensiveness when stinging mice compared to membrane-covered beakers. We assumed the mouse, possessing chemical stimuli similar to a natural predator, would represent a greater threat than the non-biological (albeit unfamiliar) surface of the beaker membranes. We predicted scorpions presented mouse targets would be more likely to issue a sting and deliver more stings with reduced latency, greater duration, and a higher percentage of wet stings than those presented beakers.*

## 2. Results

We presented threatening targets, either a dead mouse or a membrane-covered beaker, to scorpions in two separate experiments and assessed five defensive variables: sting use, latency to sting (s), number of stings before fleeing, contact duration of first sting (s), and percentage of wet stings. We summarize statistical comparisons of each experiment in Table 1. We cannot rule out a seasonal effect, so the months when experiments were conducted are indicated. Sample sizes were ample (*n* = 29–31) for each experiment but reduced for two variables, contact duration and percentage of wet stings, because some scorpions failed to exhibit stings before fleeing. We tested three sex/reproductive groups in experiment 1 (mouse target), but omitted from analyses the one gravid female encountered during experiment 2.

### 2.1. Number of Presentations

Scorpions usually fled after a single prod. However, a second prod was required to induce flight in 6.5% of 31 trials in experiment 1, and 17.2% of 29 trials in experiment 2. Although these numbers were not conducive to statistical analyses, no obvious effects of sex/reproductive group, orientation (horizontal, vertical), or body size existed.

### 2.2. Sting Use and Number of Stings

Prods generally provoked one to two stings from the scorpions (Table 1). In experiment 1 (mouse), 25.8% of 31 scorpions fled without stinging, 48.4% delivered a single sting, and 25.8% issued two stings. Total stings were too few to analyze, but logistic regression of sting use as a dichotomous variable (absent/present) yielded a non-significant model (*χ*^2^ = 1.58, df = 4, *p* = 0.81, Nagelkerke *R*^2^ = 0.04), with no differences related to sex/reproduction, orientation, or body size (Table 1).

In experiment 2, the membrane-covered beakers provoked scorpions to flee more often without stinging compared to experiment 1, but those that remained issued more stings: 37.9% of 29 scorpions fled without stinging, 24.1% delivered a single sting, and the remainder issued two (20.7%), three (6.9%), four (6.9%), or five (3.4%) stings. The ANCOVA model for number of stings revealed no group or body size differences, and no interactions (Table 1). However, logistic regression for sting use (*χ*^2^ = 10.35, df = 3, *p* = 0.016, Nagelkerke *R*^2^ = 0.41) revealed a significant effect of orientation (*p* = 0.021; Table 1), with the odds ratio suggesting that scorpions positioned vertically were 2.1 times more likely to sting than those oriented horizontally. No effect of sex or body size existed in likelihood of stinging.

### 2.3. Latency to Sting After First Prod

**Figure 1:**
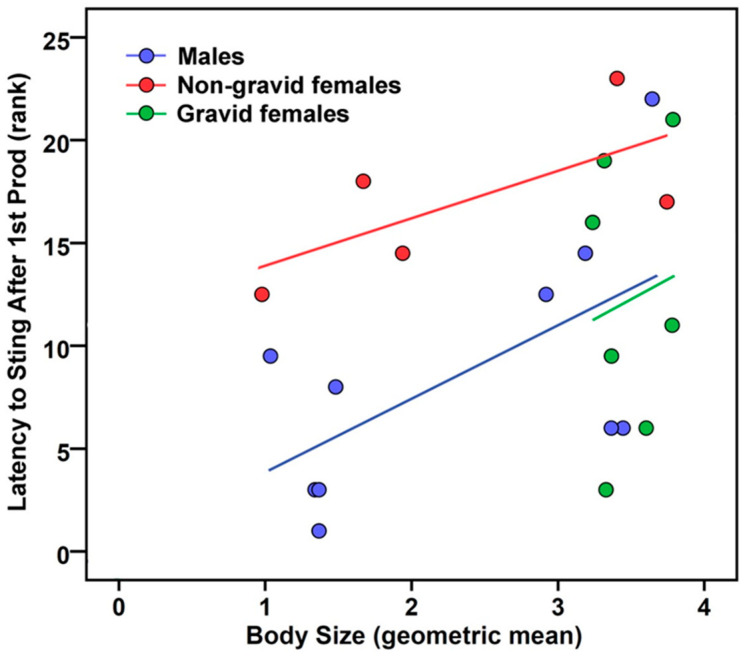
Latency of *Centruroides sculpturatus* scorpions to sting following first prod (rank-transformed data) from a dead mouse in experiment 1 increased with body size (geometric mean of 13 characters) and differed among sex and reproductive groups, with non-gravid females > males = gravid females (*n* = 5, 10, and 8, respectively). Coefficients of determination (*r*^2^) for regression slopes: males 0.36, non-gravid females 0.47, gravid females 0.02.

Scorpions that stung usually did so almost immediately (<1 s) following the first prod (Table 1). In experiment 1, the ANCOVA model suggested that non-gravid females (*p* = 0.039, partial η^2^ = 0.35) and larger individuals (*p* = 0.047, partial η^2^ = 0.24) were slowest to react by stinging the mouse (Table 1; Figure 1). Estimated marginal means (±1 S.E.) indicated that non-gravid females (0.30 ± 0.04 s) took twice as long to respond as males (0.16 ± 0.03 s) and gravid females (0.15 ± 0.05 s) at a geometric mean of 2.70 for body size. Orientation proved to be non-significant.

In experiment 2, the ANCOVA model revealed no significant group or body size differences, and no interactions (Table 1).

### 2.4. Contact Duration of Stings

In experiment 1, stinger contact with the dead mouse averaged 0.15 ± 0.03 s (range 0.02–0.60 s) for the first sting. The large effect of sex (*p* = 0.071, partial η^2^ = 0.30) in the ANCOVA model suggested gravid female stings (mean = 0.09 s) averaged half the duration of those of males (0.17 s) and non-gravid females (0.20; Table 1). No effects of orientation or size were evident, and no interactions existed. For the eight scorpions from which we obtained measurements of two stings, mean (± 1 S.E.) contact duration was similar for first and second stings (0.14 ± 0.07 and 0.13 ± 0.03 s, respectively; *t* = 0.17, df = 7, *p* = 0.87; Cohen’s *d* = 0.09).

In experiment 2, stings of the membrane-covered beaker averaged more than twice the duration (0.40 ± 0.29 s; range 0.07–0.88 s) of stings directed toward mice. However, no effects of sex, orientation, or size were evident, and no interactions existed (Table 1). For the 13 scorpions with data on two stings, contact duration was 2.4 times greater for the first sting compared to the second sting (0.30 ± 0.08 and 0.12 ± 0.02 s, respectively; *t* = 2.26, df = 12, *p* = 0.044; 95% CI of difference: 0.006–0.35 s; Cohen’s *d* = 0.88).

### 2.5. Percentage of Wet Stings

Venom expulsion was determined only for the membrane-covered beakers of experiment 2. Scorpions expended venom in most (88.2%) of the 17 trials (Table 1). However, no differences existed between the sexes or orientations.

### 2.6. Mouse Versus Beaker Targets

Because mice and beakers were presented in separate experiments, we could not directly compare scorpion responses to the two targets statistically. Consistent with the expectation that scorpions perceived mice as a greater threat than the beakers, scorpions presented mice more often fled immediately after the first presentation (93.5% vs. 82.8% of trials, respectively, as indicated above) and more often stung the mice (74.2% vs. 62.1% of trials). An interaction between orientation and target may have also existed, with mice eliciting greater sting responses among scorpions on horizontal surfaces (71.4 vs. 38.5%, respectively) and beakers provoking more stings than mice on vertical surfaces (81.3% vs. 68.4%, respectively. However, in contrast to our predictions, scorpions presented mice delivered somewhat fewer stings (mean ± 1 S.E.: 1.0 ± 0.1 vs. 1.3 ± 0.3 per trial) with greater latency to sting (0.60 ± 0.33 [with positive skew] vs. 0.29 ± 0.05 s) and briefer sting contact duration (0.15 ± 0.03 vs. 0.40 ± 0.29 s, as indicated above).

### 2.7. Relative Mass of Male and Female Scorpions

**Figure 2:**
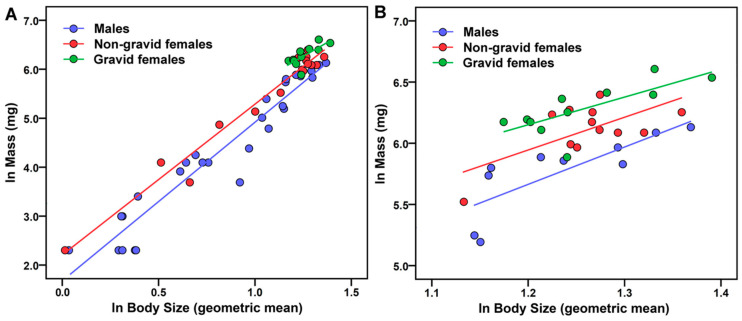
(**A**) Mass of *Centruroides sculpturatus* (log transformed data) increased with body size (geometric mean of 13 body components) and differed among sex and reproductive groups, with gravid females > non-gravid females > males (*n* = 11, 17, and 25, respectively). Coefficients of determination (*r*^2^) for regression slopes: males 0.93, non-gravid females 0.96, gravid females 0.54. (**B**) The same relationships held when considering only the largest (geometric mean > 1.1) scorpions (*n* = 11, 12, and 10, respectively). *r*^2^ values: males 0.64, non-gravid females 0.43, gravid females 0.54.

There was a positive relationship between mass and body size for all sexes and reproductive groups (Figure 2A). Female scorpions were more massive than male scorpions, with gravid females being even more massive than non-gravid females, at the same body size. Gravid females were present only in large body sizes (geometric mean > 1.1), and the same relationships held when considering only the largest scorpions (Figure 2B).

## 3. Discussion

We presented two types of targets (dead mice and membrane-covered beakers) to *C. sculpturatus* scorpions under natural environmental conditions to test four hypotheses regarding antipredator behaviors and venom expenditure. Our results failed to provide consistent support for any of the hypotheses. Scorpion reliance on defensive stinging and venom expulsion appeared to be largely independent of the hypothesized effects of (1) sex/reproductive group, (2) vertical vs. horizontal orientation, (3) body size, and (4) potential differences in threat perception arising from the two targets. Here, we discuss the relevance of findings for each hypothesis.

### 3.1. Sex Differences (Hypothesis 1)

We hypothesized that females, and more so gravid females, would, by virtue of their heavier bodies (Figure 2), rely to a greater extent on stinging and venom expulsion than males. However, only two of eight statistical comparisons yielded significance, and neither of these supported our hypothesis. Statistical differences among the three sex/reproductive groups in the mouse trial implied some behavioral variation, with non-gravid females taking the longest to react to a threat (longer sting latency), and gravid females exhibiting the briefest sting duration. These behaviors contrasted markedly with experimental findings for *C. vittatus* in a laboratory setting [23,25], in which females (mostly gravid) were more likely to attack with a shorter latency to react than males, and stung more rapidly and more often. Both *C. vittatus* [23,25,43] and *C. sculpturatus* (our unpublished data) exhibit sexual dimorphism consistent with many scorpion taxa [44], with females having shorter, stouter metasomas and larger mesosomas than males (see also [45] for other intersexual comparisons). Thus, sexual differences in sting and venom use might be expected of both species. The species differences, however, may be an artifact of different testing conditions (e.g., field vs. laboratory trials, time of day, stimulus presentation, acclimation to handling). Sexual differences in sting use by adult *T. pusillus* varied markedly with body part contacted and time of day tested [26], and the same may apply to *Centruroides* scorpions.

### 3.2. Vertical Versus Horizontal Orientation (Hypothesis 2)

We hypothesized that scorpions on horizontal surfaces would be more likely to sting and use their venom than those on vertical surfaces because the latter offers more escape trajectories. We readily located and sampled scorpions in both vertical and horizontal substrates during sweeps across assorted microhabitats (ground, boulders, block walls, trees) in the yards of a human residential area. We obtained a similar number of samples (horizontal = 27, vertical = 35) for both orientations, suggesting that the scorpions used both surface orientations readily, as observed in *C. vittatus* [33,39].

The results again failed to support our hypothesis. Only one of eight statistical comparisons yielded significance. In contrast to our hypothesis, scorpions on a horizontal surface were less likely to sting the target in the beaker trial than those on a vertical surface (38.5 and 81.3%, respectively). Scorpions in the mouse experiment were equally likely to sting from either orientation (71.4% and 68.4%, respectively). Under the conditions of testing, we suspect that orientation exerts little influence on risk assessment and likelihood of venom usage.

### 3.3. Body Size Variation (Hypothesis 3)

We hypothesized that as body size increased, scorpions would more frequently and more quickly rely on stinging and venom use because smaller scorpions have a reduced capacity to deliver effective stings. Only one of seven statistical evaluations attained significance. In contrast to our hypothesis, smaller scorpions in the mouse experiment were quicker to sting (Figure 1).

Two prior studies of scorpions also failed to support our hypothesis. Smaller individuals of *T. pusillus* were more likely to flee when body parts were restrained by tweezers (especially at night), but those that remained issued as many or more stings as the adults (Table 2 of [26]). Moreover, juvenile *H. arizonensis* prodded by a membrane-covered cup were more reactive than larger individuals, delivering wet stings with greater frequency [21]. A study of *Latrodectus hesperus* spiders, however, supported our hypothesis, with juveniles transitioning from evasive behaviors to more frequent biting and presumed venom expulsion as they grew larger [46]. In addition to inherent capacities for defense and venom use (e.g., agility, venom supply, venom potency), larger scorpions may also perceive the mouse presentation as less of a threat than the smaller scorpions do. Surface area-to-volume does not scale evenly during animal growth. The prod may be less intense to a larger scorpion because the size difference between the stimulus and subject is smaller. Moreover, the same force from the stimulus is distributed across a larger surface area of the larger subject.

Experience may also influence decision-making. During repeated exposure to predator cues, juvenile wolf spiders (*Rabidosa rabida*) became increasingly sensitized [47] and adult widow spiders (*L. hesperus*) became acclimated and less defensive [46]. Older, and therefore larger scorpions will likely have more experience with potential predators. Their experience may alter antipredator strategies resulting in a longer latency to assess risk and decide which strategy to employ.

### 3.4. Target Differences and Threat Assessment (Hypothesis 4)

We hypothesized that, because scorpions are capable of risk assessment [13,14,17,19,21,31], the mouse target would elicit more stings of longer duration than the beaker stimulus. The mouse and beaker both stimulated the subjects with downward pressure, but the mouse was assumed to offer additional proxy predator cues, specifically olfactory. Although we did not compare the two trials statistically because they were conducted at separate times without randomization, the mouse target appeared to receive a sting response more often than the beaker target (74.2% vs. 62.1% of trials, respectively), as expected, but with fewer stings per trial (68.4% vs. 81.3%, respectively), in contradiction to our expectation. However, the potential interaction between orientation and target suggested that mice elicited a greater sting response from scorpions on horizontal surfaces (71.4 vs. 38.5%, respectively), which offers some, albeit weak support for our hypothesis. Nevertheless, contact duration of the first sting was much shorter for the mouse experiment, in contrast to our expectation.

These comparisons suggest that scorpions may be assessing different levels of risk, but they are less than ideal because both targets—a thawed dead laboratory mouse and a membrane-covered beaker—incorporated novel features that may have confounded scorpion decision-making. Like many predator-response studies, we chose novel targets over those that might be encountered naturally for ethical and practical reasons. But should we expect scorpions to respond to simulated and actual predators similarly? Decision-makers are continually confronted with uncertainty (novel, incomplete, unreliable, overly abundant and complex, or conflicting cues) and may need to treat novel objects as threats [48,49,50,51]. Aversion toward novel cues, including unfamiliar spaces, situations, organisms (conspecific and heterospecific), food, fluids, and other items, is called neophobia. Numerous studies, including some involving invertebrates, suggest that selection favors neophobia responses in addition to predator responses because of the high potential cost of failure to detect a predator [48,49,50,51]. However, a review of 211 studies concluded that cues from simulated predators generate smaller effect sizes than those from actual predators [52]. Moreover, a study of amphibian tadpoles suggested that non-native predators—like the laboratory mouse we used—elicit weaker responses than native predators [53]. Until further study suggests otherwise, it seems prudent to treat novel threats presented to scorpions as reasonable proxies of natural predators with the expectation that stronger responses with greater tactical divergence might exist in real life.

The defensive tactics we assessed may not all align in favor of delivering increasing amounts of venom, as assumed with our hypotheses. Some tactics may be well-suited to deliver more venom whereas other tactics may be better suited for escape. The scorpions we tested seemed inclined to deliver stings, but they were generally few and brief before the scorpion quickly retreated. These behaviors comport with the strategy used by *C. sculpturatus* to briefly stun grasshopper mice, which are protected from venom toxicity but not momentary pain, to facilitate escape before the mouse recovers [54]. Since sting duration is related to venom volume [36], scorpions may be delivering less venom, but enough to momentarily deter their predator and facilitate their escape.

### 3.5. Wet Versus Dry Stings and Venom Metering

Venom metering is defined as the innate capacity to control or modulate the quantity of venom quantity expulsed [1,2,3,4,5,6]. This capacity differs from venom boluses that are consistent with each discharge (the bullet hypothesis) or vary because of extrinsic factors beyond the animal’s control [5]. Unfortunately, recent studies have conflated venom metering with selective sting use (whether or not to deliver a sting) and venom heterogeneity (changes in venom composition with successive expulsions). Because the quantity of venom expulsed during a sting or bite can theoretically range from none to the full content of the venom glands, we consider a kinematically normal sting or bite, including creation of a wound (which is required of venom delivery [55]), to be an act of venom metering. Dry stings may comprise an effort to withhold a limited and costly commodity when the startle effect or the pain from wound generation might suffice to ward off a predator.

Dry stings have been reported in prior laboratory-based studies of scorpion venom expenditure [13,16,17,20,21,22]. The proportion of dry stings decreased with greater levels of threat in *Parabuthus transvaalicus* [13] but was unrelated to threat in *T. stigmurus* [16]. The proportion of dry stings by *H. arizonensis* increased during 10 consecutive stings, but did not result from venom depletion [20]. Our studies of *C. sculpturatus* (here) and *S. mesaensis* [17] comprise the first effort to our knowledge of quantifying dry sting frequency in wild scorpions. The frequency of dry stings delivered to the beaker was less for *C. sculpturatus* (11.8%) compared to *S. mesaensis* (47.6%), suggesting greater reliance on venom by the former species [17]. Presumably dry stings have been reported in human cases of scorpionism, with frequency estimates ranging wildly from 1% [56] to 90% [57]. Such estimates are complicated because those experiencing dry stings may not show up at clinics (hence, underreported) and no envenomation may be difficult to distinguish from mild envenomation (hence, overreported). For stings delivered experimentally to human fingers in the field by *S. mesaensis*, dry stings were more frequent with simple finger taps to the body (77.8%) compared to pressing down on the scorpion’s body (33.3%), which clearly indicated a role of threat perception in venom deployment [17].

Venom composition can change with venom gland depletion across successive scorpion stings [58], but the change is directly related to quantity of venom expulsed [13] and state of venom regeneration [9,10]. Thus, scorpions do not directly regulate the composition of venom that emerges during successive stings, and evidence of composition change (from clear to milky in appearance) does not constitute venom metering. A prior study presented evidence for adaptive plasticity in venom composition that resulted from repeated exposure to predators [59]. However, the study design confounded exposure to a surrogate predator with frequency of stings, and therefore the composition differences relative to naïve scorpions not exposed to predators could have been attributed to the direct effects of venom depletion and regeneration.

### 3.6. Unaccounted Variation in Environmental Conditions

Experiments conducted in the field may be better for assessing the natural behavior of animals, but unaccounted variation in environmental conditions may obfuscate divergent behavioral tactics. Environmental conditions that might influence defensive behavior include habitat structure, proximity to shelter, body temperature, light intensity (including that of the UV flashlight), and proximity to other scorpions. Here, we briefly address a few of these that are known to influence scorpion behavior.

Visible light [60,61], light of different wavelengths [62,63], and temperature [24,56,64,65] are all recognized as having possible influences on scorpion behavior. At optimum temperatures, scorpion species show peak performance: *C. vittatus* scorpions can sting more frequently and accurately and flee quicker and farther [24], and *Paruroctonus marksi* can sprint quicker [66]. In *Buthus atlantis*, researchers observed increased venom expenditure and protein concentration at optimum temperatures [19]. Although temperatures were similar among nights during our experiments, the scorpions rested in different microhabitats throughout the day, which potentially affected body temperatures during testing. A scorpion that sought refuge in the walls of a home or a backyard fountain during the day may have had a different body temperature, and therefore altered fleeing and stinging, than one that emerged from under a brick path baking in the heat of the desert day. Cooper noted that males of the lizard *Eumeces laticeps* allowed closer approach before fleeing when in the presence of females than when alone [28]. In our study, we did not record proximity of neighboring scorpions to assess potential social buffering, the reduction in stress when in close proximity to a companion. Social buffering has been reported in another venomous taxon, the rattlesnake *Crotalus helleri* [67]. *Centruroides* scorpions often aggregate, in contrast to many other scorpion taxa [24].

The invasive population we studied has successfully established itself in suitable habitat that offers niches that we judged similar to those in its native habitat. For this reason, we considered the environmental conditions to be natural. Nevertheless, a number of artificial components (e.g., electric lights, swimming pools, turf) and a high prevalence of predators (domesticated cats and dogs) may have added variance to the behavioral responses. Some invasive populations of invertebrates exhibit very different behavior than native populations [68]. This type of study might benefit from controlled experimental trials with two samples of scorpions, one from the introduced population and the other from a native population.

## 4. Conclusions

Our study failed to support the first three hypotheses regarding the influences of sex, substrate orientation, and body size on risk assessment, defensive stinging, and venom deployment. The few statistically significant comparisons with large effect sizes contradicted rather than supported our hypotheses. The fourth hypothesis received, at best, mixed support from anecdotal comparisons, as the data were not amenable to statistical analyses. Our field-based findings contrast with those of previous laboratory-based studies that have demonstrated the existence of sex-, age-, and threat-related differences in the defensive behaviors and venom use of scorpions. We suspect that scorpion behavior in our field study was influenced by unaccounted environmental factors that rendered the influences of sex, substrate orientation, body size, and target threat unclear. Nevertheless, the behaviors we documented provide a better understanding of the range of behaviors exhibited by *C. sculpturatus* under natural environmental conditions.

## 5. Materials and Methods

### 5.1. Ethics Statement

This study complied with expectations of the Institutional Animal Care and Use Committee of Loma Linda University, which deemed no protocols or permits were necessary for invertebrate research and handling. Research met the standards set forth by the Office of Research Affairs and by the Faculty of Graduate Studies. *Centruroides sculpturatus* is not an endangered or protected species, and the population we studied is invasive to the region. No permits were needed, but consent to handle and remove specimens was received from property owners through the Coachella Valley Mosquito and Vector Control. Efforts were made to minimize suffering of all animals. Specimens were euthanized in accordance with current standards for arachnids [69,70] within 5 h of capture.

### 5.2. Defensive Behavior Trials

We studied an introduced population of bark scorpions in a private residential community in Indio, California, during June through September 2019. We began field observations 30–60 min after sunset using ultraviolet (UV, ~495 nm) flashlights to locate scorpions [71]. After locating a scorpion, we approached with indirect UV light until positioned to conduct the trial, as scorpions can respond to UV light [72].

**Figure 3:**
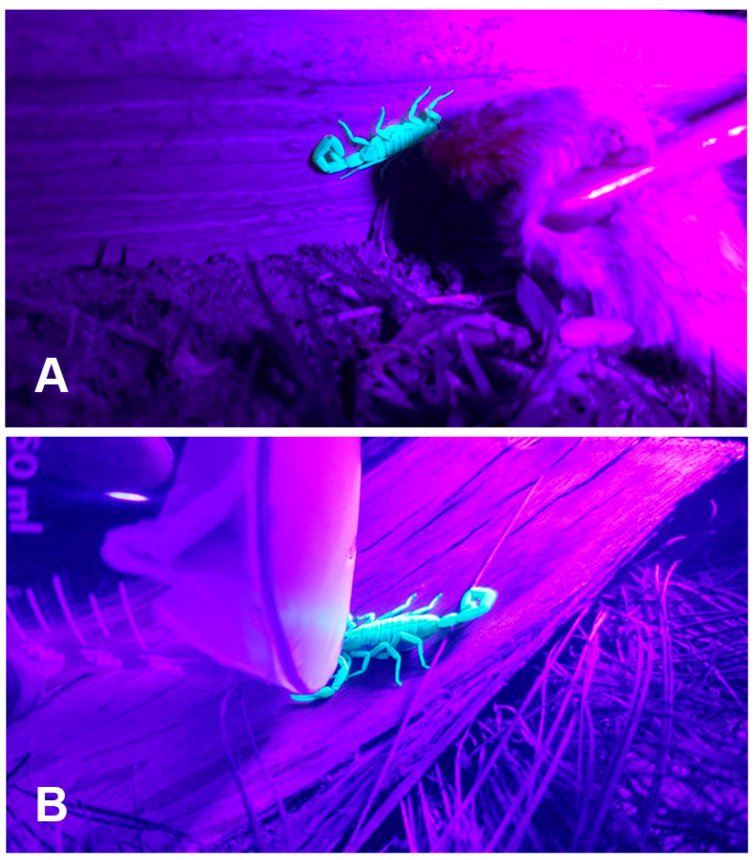
Anterior presentation of two stimulus conditions to *Centruroides sculpturatus* scorpions. Brief prods (up to 5 s) contacting the prosoma were repeated, if necessary, until the scorpion fled at least 30 cm from its original position. Scorpions were tested where encountered on both horizontal and vertical surfaces. (**A**) Dead adult mouse (*Mus musculus*) presented via forceps to a scorpion on a vertical block wall. (**B**) Membrane-covered 150-mL glass beaker presented by hand to a scorpion on a horizontal palm frond.

We subjected scorpions (*n* = 61) to one of two stimuli (Figure 2) in two separate experiments. In experiment 1, we presented a dead (freshly thawed) laboratory mouse (*M. musculus*), purchased frozen from a local rodent breeder, via 37 cm forceps to simulate a natural predator (c.f. [59]), the grasshopper mouse (*Onychomys* spp.), that *C. sculpturatus* encounters in its natural range [73]. For this experiment, we could not ascertain whether scorpion stings were accompanied by venom expulsion. Thus, for experiment 2, we presented a Parafilm^TM^-covered 150-mL beaker by hand to simulate a predation threat and to visualize venom expulsion. Each trial entailed briefly contacting the scorpion with the target from an anterior approach on the dorsal prosoma surface for 3–5 s to provoke a response. If there was no response to the prod, we reapplied the stimulus after a 1 s pause, repeatedly if necessary, until the scorpion undertook evasive movements of at least 30 cm from its initial position. We videotaped all interactions at a posterolateral angle from a distance of 15–30 cm (Figure 3) using a cell phone set to 30 (experiment 1) or 60 fps (experiment 2) and illuminated only by UV light.

### 5.3. Morphological Measurements

**Figure 4:**
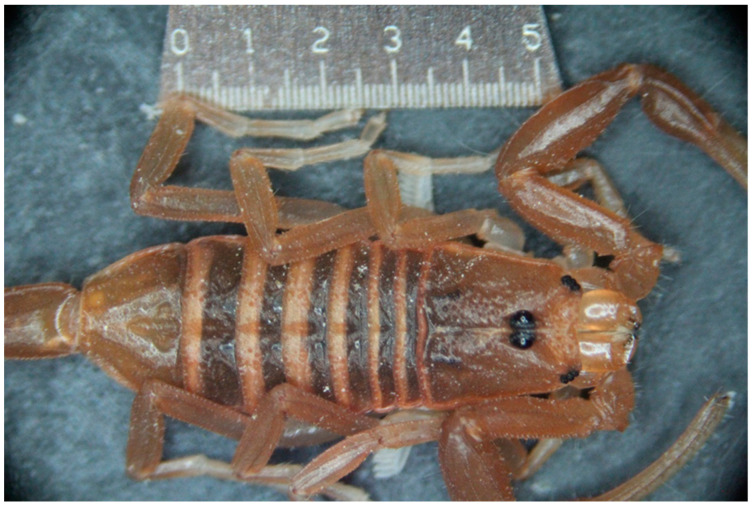
Representative photograph of *Centruroides sculpturatus*, including millimeter scale to calibrate measurements in ImageJ software.

Upon completing each behavioral trial, we captured the scorpion for subsequent euthanasia and storage in a −20 °C freezer. We visually identified sex based on pectine length and orientation and metasoma length. We determined female gravid status by inspecting the mesosoma’s partially translucent pleural membrane on the ventral side for the presence of embryos. We photographed the thawed specimens using a Nikon SMZ-10A microscope (Nikon Corporation, Tokyo, Japan) and a Canon EOS60D camera (Canon Inc., Tokyo, Japan), with a small scale (5 mm with 0.1 mm divisions) included for calibration (Figure 4).

We used the photographs to measure 13 morphological components and total length using the Fiji software package of ImageJ (version 1.52p, https://imagej.nih.gov/ij/; accessed 30 December 2019). The body components included prosoma length and width; mesosoma length; right pedipalp length, width, and height; metasoma segments 1 and 5 length and width; telson width and height; and telson-to-subacular groove (excluding stinger) length. Three measurements were taken for each component and then averaged. We computed geometric mean (*n*th root of the product of *n* measures) using the 13 morphological components measured for overall body size, which provides an unbiased measure of overall body size [74,75]. Because this approach requires measurement of multiple body components, we suspect it is the least biased measure of overall body size (c.f. [42,76]). Mass of frozen scorpions was obtained using a precision balance.

### 5.4. Behavioral Measurements

During field-by-field video review using Kinovea (www.Kinovea.org; accessed 30 December 2019) open-source software (version 0.8.15), we recorded the following dependent measures: total number of (1) prods received and (2) stings exhibited (defined as telson making physical contact) until the scorpion fled sufficiently to terminate the trial; (3) latency to sting after the first prod (nearest 0.017 s); and (4) duration of contact for the first sting (nearest 0.017 s). In the field, we also recorded (5) the substrate the scorpion was on (e.g., block wall, palm tree, ground), including its vertical or horizontal orientation, and (6) whether stings to membrane-covered beakers were dry or wet (visible venom droplets).

### 5.5. Analyses

We conducted all analyses and produced the scatterplots in Figure 1 and Figure 2 using IBM SPSS Statistics for Windows ver. 23 (IBM Corp., Armonk, NY, USA) with alpha set to 0.05. We analyzed data only within each experiment, and not between experiments. We screened data to verify assumptions of normality (Shapiro–Wilk test), homoscedasticity (Levene test), and linearity (bivariate scatterplots) were met. Continuous dependent measures sometimes failed to meet parametric assumptions, but rank-transformation consistently improved the data distribution, and in several tests yielded significance when original data only approached significance. Thus, we report analyses of ranks for latency to sting and duration of sting contact. We set alpha to 0.05, and chose not to adjust it (with Bonferroni corrections) or the *p*-values (with false discovery rate) for multiple tests [77] because doing so, especially with small sample sizes, overemphasizes the importance of null hypothesis testing when effect size is more meaningful [78], and unacceptably increases the probability of making type II errors (i.e., the hyper-Red Queen phenomenon: the more research one does, the lower the probability that a significant result will be found [79]).

We analyzed sting use as a dichotomous variable (absent, present) in a binomial logistic regression model [80,81], treating sex/reproductive group (male, non-gravid female, gravid female), orientation (horizonal, vertical), and body size as predictors. We computed odds ratios with 95% C.I.s, and Nagelkerke *R*^2^ as an effect size, with values of ~0.01, ~0.09, and ~0.25 corresponding loosely to small, medium, and large effects, respectively. We subjected the three continuous measures (number of stings before fleeing, latency to sting after first prod, and contact duration of first sting) to analyses of covariance (ANCOVAs) [80,81], treating sex/reproductive group and orientation as independent variables and body size as a covariate. Data met the assumption of homogenous regression slopes. We computed partial eta-squared (η^2^*)* as effect sizes, with values of ~0.01, ~0.06, and ≥0.14 corresponding loosely to small, medium, and large effect sizes, respectively [82]. We conducted paired *t*-tests [81] to compare duration of contact for first and second stings, and assessed effect sizes via Cohen’s *d*, with values of ~0.2, ~0.5, and ~0.8 corresponding loosely to small, medium, and large effects, respectively [82]. For the proportion of wet stings, sample sizes were insufficient to use logistic regression, so we used Fisher’s exact test to compare percentage of wet stings between the sexes and between orientation categories, with phi (ϕ) values of ~0.1, ~0.3, and ~0.5 corresponding to small, medium, and large effects, respectively [82].

We analyzed the relative mass of scorpions using an ANCOVA as well, with sex/reproductive group as an independent variable and body size as a covariate. Data met the assumption of homogenous regression slopes. We computed effect sizes as described above for the prior ANCOVA models.

## Figures and Tables

**Table 1:** Summary of antipredator behaviors (percent or mean ± 1 S.E. followed by *n*) exhibited by *Centruroides sculpturatus* scorpions in response to two different stimulus conditions: a dead mouse in experiment 1 and a membrane-covered beaker in experiment 2. The subjects were either on a horizontal or vertical substrate. Significant or especially large effects are indicated by bold font.

Dependent Measures and Main Effects ^a^	Experiment 1 Mouse Trials (June–July)	Experiment 2 Beaker Trials (July, September)
**Sting use (%)**		
Males	71.4 (14)	52.9 (17)
Non-gravid females	83.3 (6)	75.0 (12)
Gravid females	72.7 (11)	—
Horizontal orientation	71.4 (14)	38.5 (13)
Vertical orientation	68.4 (19)	81.3 (16)
Main effect of sex	*p* = 0.709 ^b^	*p* = 0.090, OR = 0.67–252.90 ^b^
Main effect of orientation	*p* = 0.668, OR = 0.09–4.76 ^b^	***p* = 0.021, OR = 1.62–314.40 ^b^**
Main effect of size	*p* = 0.321, OR = 0.57–5.48 ^b^	*p* = 0.701, OR = 0.16–3.37 ^b^
**Number of stings before fleeing**		
Males	1.1 ± 0.2 (14)	0.9 ± 0.3 (17)
Non-gravid females	1.0 ± 0.3 (6)	1.8 ± 0.5 (12)
Gravid females	0.9 ± 0.2 (11)	—
Horizontal orientation	0.9 ± 0.2 (14)	1.0 ± 0.5 (13)
Vertical orientation	1.0 ± 0.2 (19)	1.6 ± 0.3 (16)
Main effect of sex	—	*p* = 0.136, η^2^ = 0.09 ^c^
Main effect of orientation	—	*p* = 0.221, η^2^ = 0.06 ^c^
Main effect of size	—	*p* = 0.975, η^2^ < 0.01 ^c^
**Latency to sting after first prod (s)**		
Males	0.21 ± 0.11 (10)	0.31 ± 0.08 (9)
Non-gravid females	1.77 ± 1.47 (5)	0.28 ± 0.08 (8)
Gravid females	0.36 ± 0.13 (8)	—
Horizontal orientation	0.98 ± 0.75 (10)	0.32 ± 0.06 (5)
Vertical orientation	0.31 ± 0.09 (13)	0.28 ± 0.07 (12)
Main effect of sex	***p* = 0.039, η^2^ = 0.35 ^c^**	*p* = 0.827, η^2^ < 0.01 ^c^
Main effect of orientation	*p* = 0.817, η^2^ < 0.01 ^c^	*p* = 0.473, η^2^ = 0.04 ^c^
Main effect of size	***p* = 0.047, η^2^ = 0.24 ^b^ (+)**	*p* = 0.638, η^2^ = 0.02 ^c^
**Contact duration of first sting (s)**		
Males	0.17 ± 0.07 (10)	0.38 ± 0.09 (9)
Non-gravid females	0.20 ± 0.05 (5)	0.36 ± 0.12 (8)
Gravid females	0.09 ± 0.01 (8)	—
Horizontal orientation	0.14 ± 0.05 (10)	0.42 ± 0.15 (5)
Vertical orientation	0.15 ± 0.04 (13)	0.35 ± 0.08 (12)
Main effect of sex	***p* = 0.071, η^2^ = 0.30 ^c^**	*p* = 0.749, η^2^ = 0.01 ^c^
Main effect of orientation	*p* = 0.253, η^2^ = 0.09 ^c^	*p* = 0.736, η^2^ = 0.01 ^c^
Main effect of size	*p* = 0.639, η^2^ = 0.02 ^c^	*p* = 0.649, η^2^ = 0.02 ^c^
**Percentage of wet stings (%)**		
Males	—	87.5 (8)
Females	—	88.9 (9)
Horizontal orientation	—	100.0 (4)
Vertical orientation	—	84.6 (13)
Main effect of sex	—	*p* = 1.000, ϕ = 0.02 ^d^
Main effect of orientation	—	*p* = 1.000, ϕ = 0.20 ^d^

^a^ Sex included three groups: males, non-gravid females, gravid females. ^b^ Logistic regression *p*-value and 95% C.I. of odds ratio (OR) for effect size; ORs not supplied for the three pairwise comparisons among sex groups in experiment 1 (all non-significant). ^c^ Analysis of covariance (ANCOVA) and partial η^2^ for effect size (latency to react and contact duration were rank-transformed prior to analysis); the significant association with body size is indicated as a positive relationship (+). ^d^ Fisher’s exact test and phi (ϕ) for effect size.

## Data Availability

The raw data supporting the conclusions of this article will be made available by the authors on request.

## Abbreviation

The following abbreviation is used in this manuscript:

ANCOVA	Analysis of covariance
